# Supplementary material for: Autologous Human Immunocompetent White Adipose Tissue‐on‐Chip
Source: Adv Sci (Weinh). 2022 Apr 24;9(18):2104451. doi: 10.1002/advs.202104451 (PMC9218765; doi:10.1002/advs.202104451)
Supplement: Supplementary file 1 — Supporting Information [file ADVS-9-2104451-s005.pdf]

Supplements

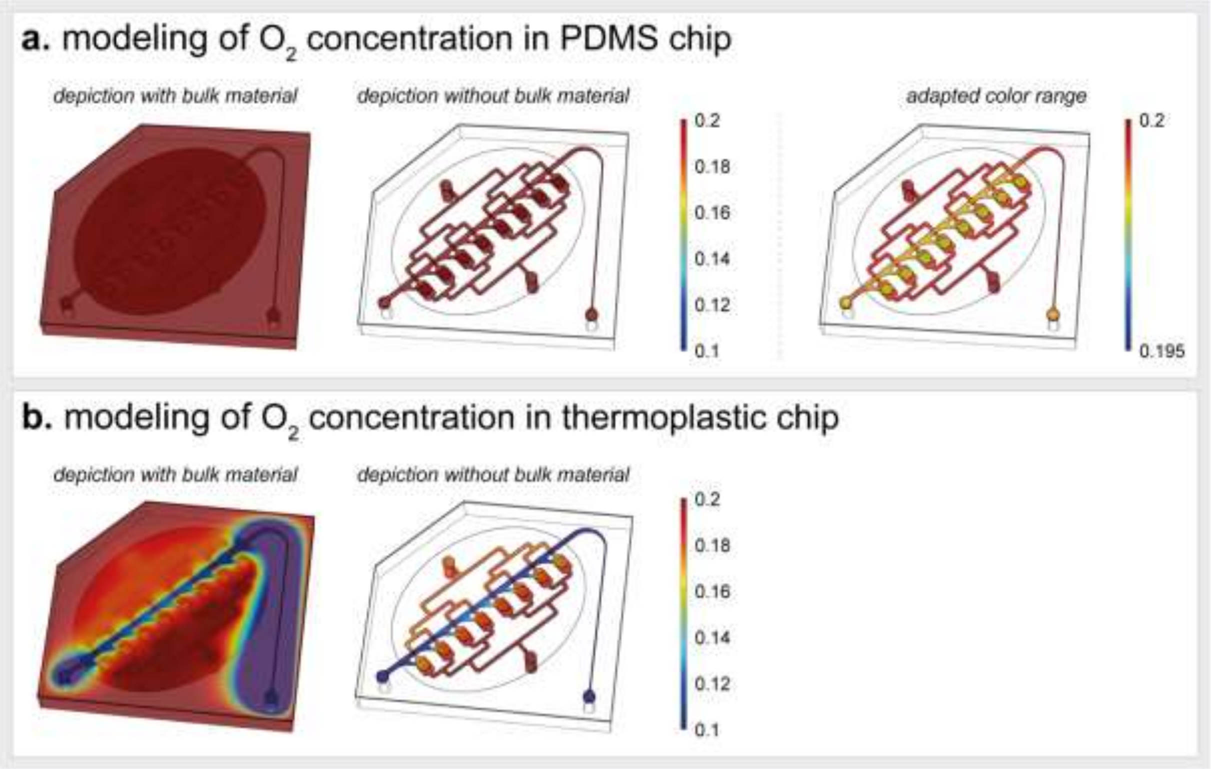

Figure S1. Simulation of on-chip O_2 availability with inclusion of O_2 consumption by cells in tissue compartment, delivery with media perfusion (20 µL/h) and diffusion through bulk material. O2 consumption was assumed for the entire tissue compartment. **a.** O_2 concentration in a microfluidic platform made from PDMS as bulk material. Adjusting of color range revealed a O_2 concentration of approximately 0.198 mol/m^3 in tissue chambers. **b.** O_2 concentration in a theoretical microfluidic platform made from a thermoplastic as bulk material. Due to lower O_2 permeability of the bulk material, the O_2 supply depends on a constant perfusion of fresh, saturated cell culture medium, which yields an O_2 concentration of approximately 0.18 mol/m^3.

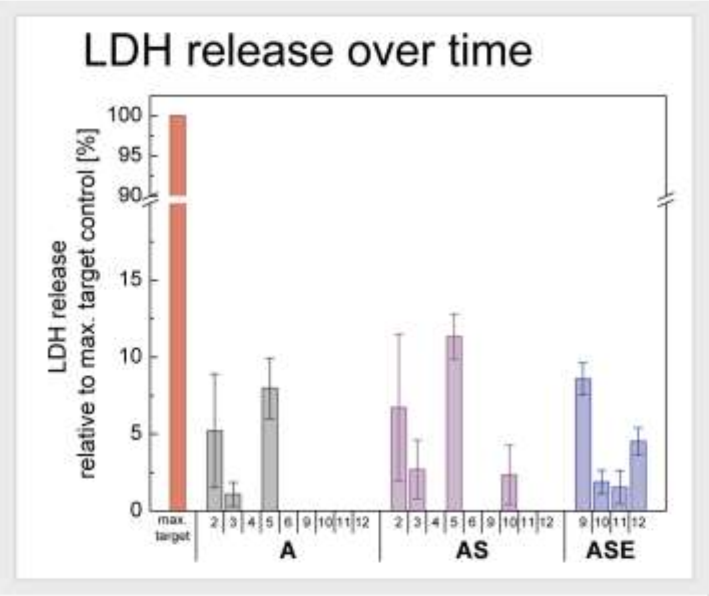

Figure S2. Cytotoxicity assessment for different culture modes during 12 d of on-chip culture. LDH release as cytotoxicity readout was determined every 24 h from media effluents. Absorbance values

normalized to a respective target cell maximum LDH release control (100%), which was determined for each culture condition individually. On days that do not show bars, LDH release into the media effluents was not detectable with the readout method. Data were pooled from 3 donors. Due to different flow conditions, days 7 and 8 were omitted from the analysis. *A*: adipocyte-only chips; *AS*: adipocyte-SVF co-culture chips; *ASE*: adipocyte-SVF-mvEC co-culture chips.

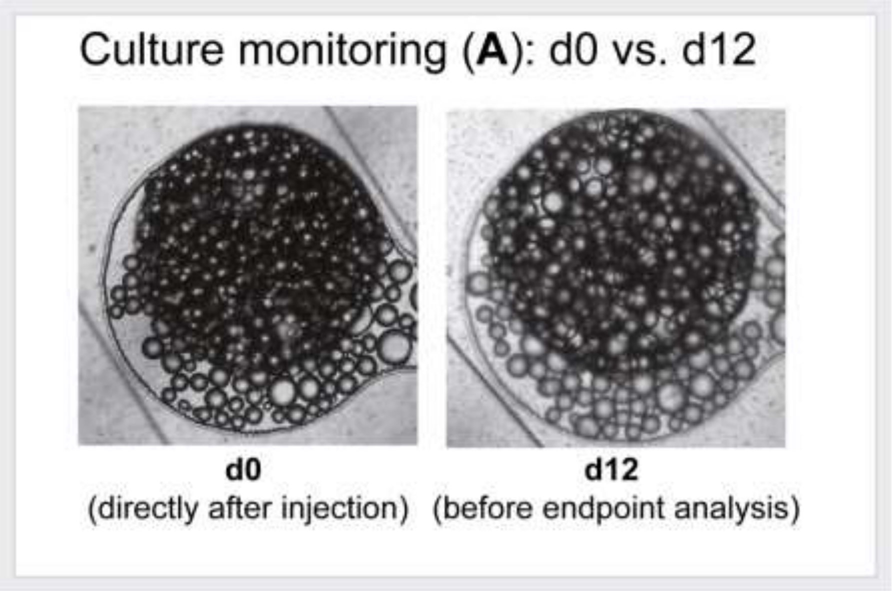

Figure S3. Monitoring of adipocytes-on-chip throughout a 12-day culture period. The same tissue chamber is shown directly after cell injection (d0) and directly prior to endpoint analysis (d12). Adipocyte morphology appeared comparable over time.

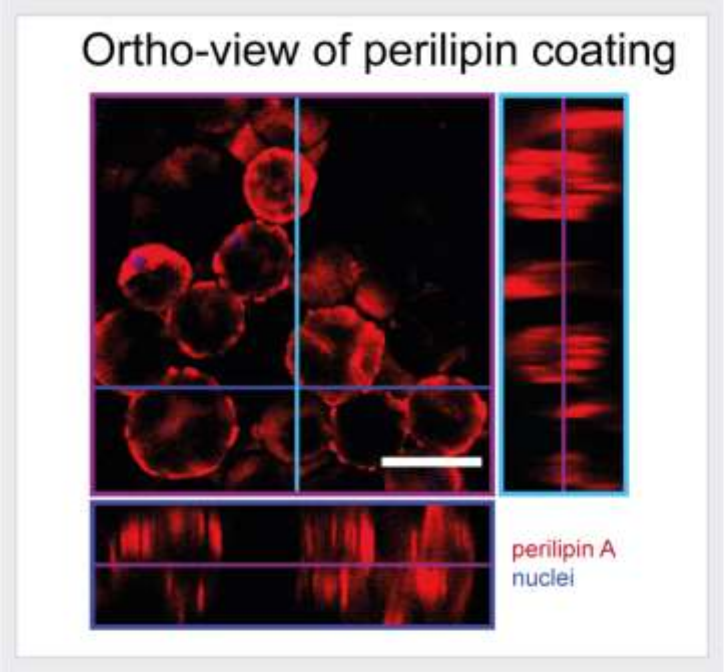

Figure S4. Perilipin A coating of adipocytes' lipid droplets represented in orthogonal views. Perilipin A clustered to microdomains on the adipocytes' lipid droplets. Scale bar equals 100 μm.

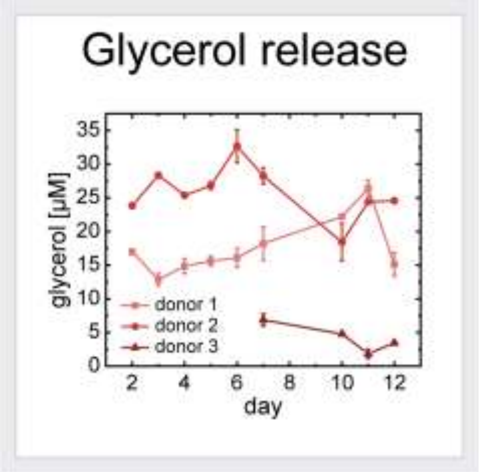

Figure S5. Monitoring of glycerol release from adipocytes into media effluents over time (donors 1 and 2 in biological duplicates; donor 3 in biological triplicates).

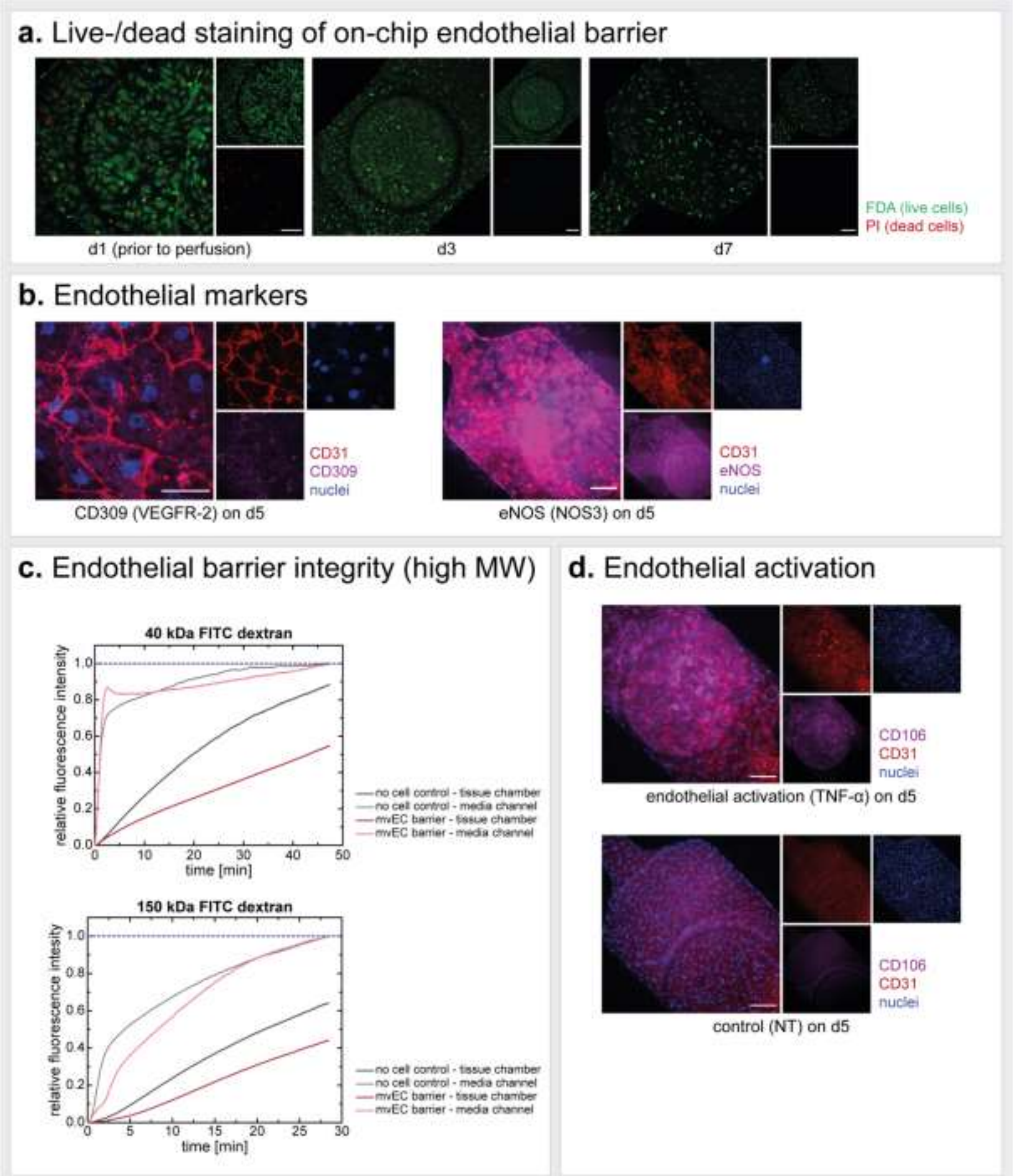

Figure S6. Additional characterization of on-chip endothelial barrier. (a) Live-/dead staining of on-chip mvEC layer on different days of analysis revealed overall high long-term viability. On d1, prior to connection of constant media perfusion, there were several dead cells (potentially not fully attached remnants from the injection process). On d3 and d7, there were only a few dead cells in between the viable monolayer. Scale bars equal 200 µm. (b) In addition to CD31, we confirmed EC identity (and indicated proper functionality) by visualizing CD309 (alternatively VEGFR-2; main receptor of VEGF and important mediator of quiescent and active endothelium) (isotype controls in Figure S10; scale bar equals 50 µm) and eNOS (alternatively NOS3; for nitric oxide production) (scalebar equals 200 µm). (c) The permeability of the endothelial barrier on the chips' membranes was assessed by using fluorescent macromolecular tracers (here: 40 kDa and 150 kDa FITC-dextrans). The running time of the performed assays was not long enough to achieve equilibria between media channel and tissue chamber fluorescence intensity; yet there are clear trends indicating differences between cellularized vs. plain membrane transport as well as between different molecular weights. (d) Visualization of

CD106 (alternatively VCAM1; expressed by activated endothelium for leukocyte-endothelial cell adhesion) for TNF- α-treated and untreated endothelial layers (scale bar equals 200 μm).

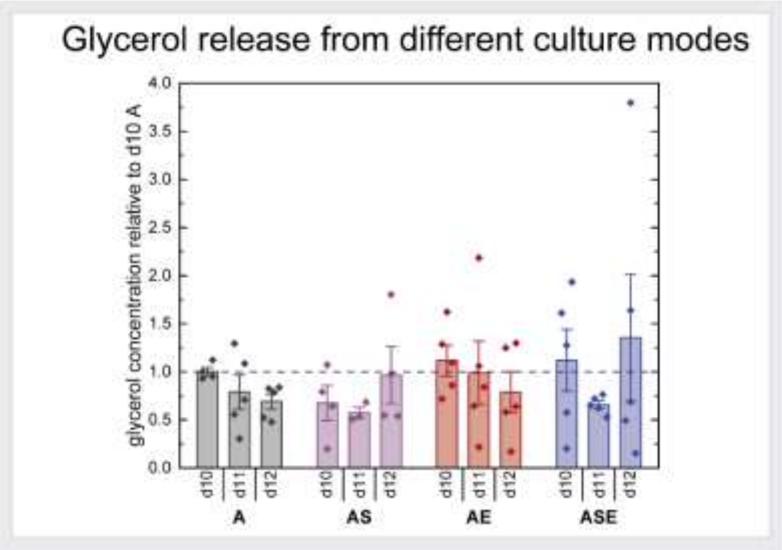

Figure S7. Normalized glycerol release over time (d10-d12) from culture modes A, AS, AE, and ASE from donor-specific cells.

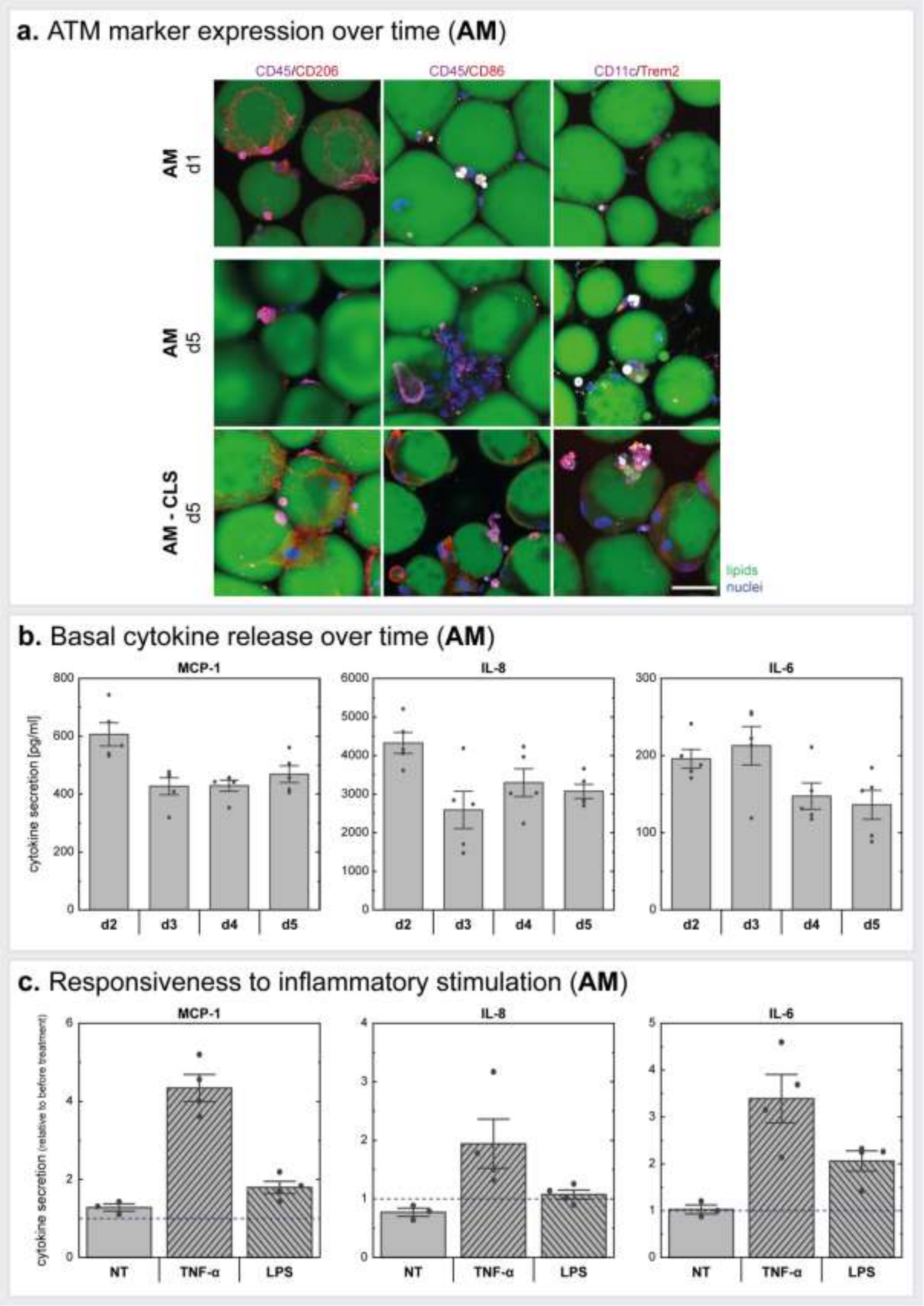

Figure S8. Characterization of AM co-cultures on chip. (a) Expression of different ATM markers over time. Imaging data revealed similar ATM marker expression at d1 and d5. Formation of individual, dispersed crown-like structures (CLS) was only observed at d5. (b) Basal release of the cytokines MCP-1, IL-8 and IL-6 was stable throughout the 5-day culture period (scalebar equals 50 μm). (c) Cytokine release of adipocyte-CD14+-cell co-culture chips in response to TNF-

α or LPS stimulation. Stimulation was performed for 24 h from d4-d5. Cytokine concentrations released throughout these 24 h were normalized to the cytokine levels determined for the 24 h before treatment for each chip.

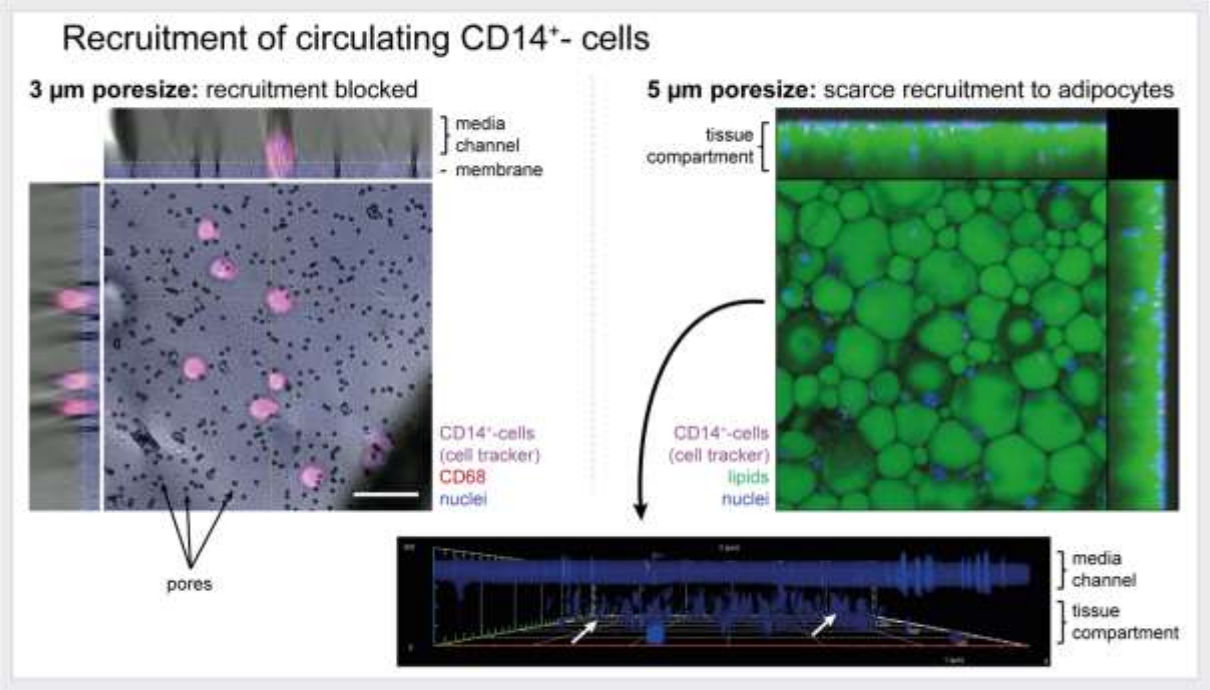

Figure S9. Comparison of recruitment of perfused CD14+-cells to adipocytes-on-chip (culture mode A) through 3 µm and 5 µm pore-sized membranes. CD14+-cells did not seem to be able to infiltrate the adipocyte chamber through 3 µm diameters pores in the chips' membranes. When building in 5 µm pore-sized membranes, a scarce recruitment into the tissue compartment could be detected as indicated by verification of cell tracker fluorescence signal in the tissue chamber.

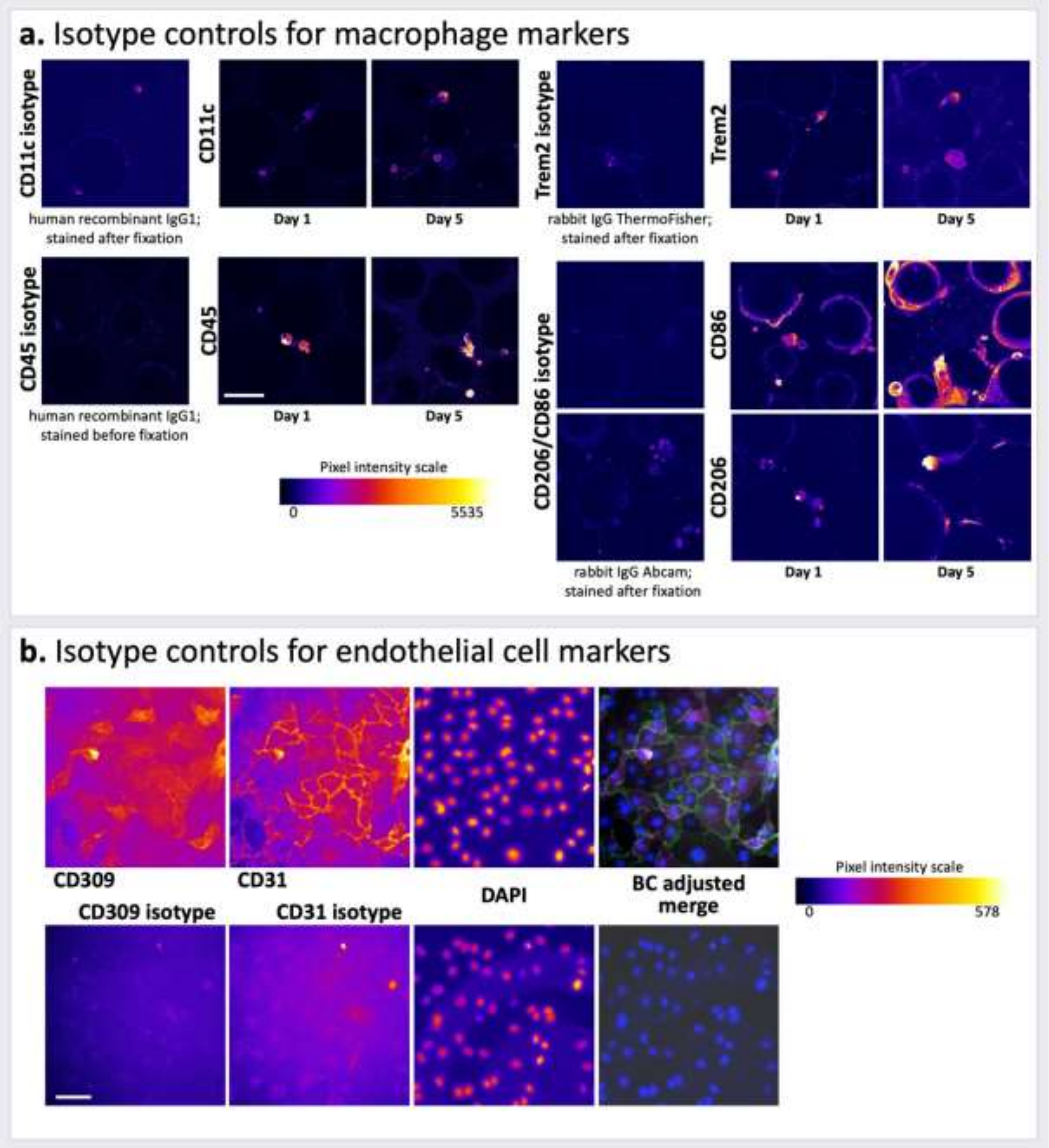

Figure S10. Isotype controls for (a) macrophage markers stained on-chip and (b) endothelial cell markers stained in well plate culture (scalebars equal 50 µm).

Table S1. Estimates of absorption into PDMS of key substances administered to, or sampled from, WAT-on-chip tissues compared to highly absorbed reference substances. Substances with a n-octanol/water partition coefficient (LogP) ≥ 2.62 (as defined by Wang et al.,[1]) combined with a small molecular weight (MW) are very likely remarkably absorbed (indicated by red highlighting). Key substances used and analyzed within the scope of the WAT-on-chip project are likely not significantly absorbed due to low hydrophobicity (i.e., low logP value) or high molecular weights (indicated by blue highlighting).

N/A: no specifications found in literature; LMW: low molecular weight; HMW: high molecular weight.

**Substance**	**logP**	**MW [g/mol]**
**Reference substances with high absorbtion into PDMS**		
Rhodamine 6G[2]	2.62	479.02
Nile red[3]	3.98	318.376
Estrogen[4]	3.67	296.41
**Media ingredients and drugs/stimulants used in this study**		
Insulin[5]	N/A	$12x10^3$<br>→ too large to fall into "small" molecule category
Rosiglitazone[6]	2.49 (predicted)	357.43
GM-CSF[7]	N/A	$16.3x10^3$
(−)-Isoproterenol hydrochloride[8]	-0.317 (predicted)	247.72
Tumor necrosis factor α[9]	N/A	$25.6x10^3$
**Metabolites/cytokines measured in this study**		
Glycerol[10]	-1.86	92
Adiponectin[11]	N/A	$180x10^3$ (LMW) or $360x10^3$ (HMW)
Leptin[12]	N/A	$16x10^3$
Adipsin[13]	N/A	$27x10^3$
RBP4[14]	N/A	$21x10^3$
MCP-1[15]	N/A	$11x10^3$
IL-6[16]	N/A	$23.7x10^3$
IL-8[17]	N/A	$11.1x10^3$
Angiopoeitin 2[18]	N/A	$56.9x10^3$
**Lipids and lipokines (not quantified in this study)**		
Hexadecanoic acid (palmitic acid)[19]	7.17	256.43
9,12-Octadecadienoic acid (linoleic acid)[20]	7.05	280.452
(Z)-Hexadec-9-enoic acid (palmitoleic acid)[21]	6.58 (predicted)	254.414

1: Wang, J. D., Douville, N. J., Takayama, S. & ElSayed, M. Quantitative Analysis of Molecular Absorption into PDMS Microfluidic Channels. Ann Biomed Eng 40, 1862–1873 (2012).
2: Toepke, M. W. & Beebe, D. J. PDMS absorption of small molecules and consequences in microfluidic applications. Lab Chip 6, 1484 (2006).
3: Wang, J. D., Douville, N. J., Takayama, S. & ElSayed, M. Quantitative Analysis of Molecular Absorption into PDMS Microfluidic Channels. Ann Biomed Eng 40, 1862–1873 (2012).
4: Regehr, K. J. et al. Biological implications of polydimethylsiloxane-based microfluidic cell culture. Lab Chip 9, 2132 (2009).
5: https://www.proteinatlas.org/ENSG00000254647-INS; date of access: 2021-12-08.
6: https://comptox.epa.gov/dashboard/chemical/details/DTXSID7037131; date of access: 2022-01-05.
7: https://www.proteinatlas.org/ENSG00000164400-CSF2; date of access: 2022-01-05.
8: https://comptox.epa.gov/dashboard/chemical/details/DTXSID6025486; date of access: 2022-01-05.
9: https://www.proteinatlas.org/ENSG00000232810-TNF; date of access: 2022-01-05.
10: https://comptox.epa.gov/dashboard/chemical/details/DTXSID9020663; date of access: 2022-01-05.

11: Lara-Castro, C., Luo, N., Wallace, P., Klein, R. L. & Garvey, W. T. Adiponectin Multimeric Complexes and the Metabolic Syndrome Trait Cluster. Diabetes 55, 249–259 (2006).
12: Münzberg, H. & Morrison, C. D. Structure, production and signaling of leptin. Metabolism 64, 13–23 (2015).
13: https://www.proteinatlas.org/ENSG00000197766-CFD; date of access: 2022-01-05
14: Steinhoff, J. S., Lass, A. & Schupp, M. Biological Functions of RBP4 and Its Relevance for Human Diseases. Front. Physiol. 12, 659977 (2021).
15: https://www.proteinatlas.org/ENSG00000108691-CCL2; date of access: 2021-12-08.
16: https://www.proteinatlas.org/ENSG00000136244-IL6; date of access: 2021-12-08.
17: https://www.proteinatlas.org/ENSG00000169429-CXCL8; date of access: 2021-12-08.
18: https://www.proteinatlas.org/ENSG00000091879-ANGPT2; date of access: 2021-12-08.
19: https://comptox.epa.gov/dashboard/chemical/details/DTXSID2021602; date of access: 2022-01-05.
20: https://comptox.epa.gov/dashboard/chemical/details/DTXSID2025505; date of access: 2022-01-05.
21: https://comptox.epa.gov/dashboard/chemical/details/DTXSID0041197; date of access: 2022-01-05.

Table S2. List of materials/reagents/media/devices/software used within the scope of the WAT-on-chip study.

**Object**	**Specifications**	**Provider**	**Stock concentration**	**Final concentration**
**General reagents and buffers**				
Bovine serum albumin (BSA)	**P6154-100GR**	VWR International GmbH, Darmstadt, Germany	-	Dependent on application; as stated in Materials and Methods section
DPBS, without calcium, without magnesium (liquid) (PBS^-)	**L0615-500**	VWR International GmbH, Darmstadt, Germany	-	-
Dulbecco's Phosphate Buffered Saline With MgCl2 and CaCl2 (PBS^+)	**D8662**	Sigma-Aldrich, St. Louis, MO, USA	-	-
**Chip fabrication and preparation**				
3M Scotchpak™ (Coated-foil)	**1022 Release Liner Fluoropolymer Coated**	3M, Saint Paul, MN, USA	-	-
Biopsy punch; 0.75 mm diameter	**504529**	World Precision Instruments, Friedberg, Germany	-	-
Coverslips	**N-21-000627**	Langenbrinck GmbH, Emmendingen, Germany	-	-
ipCELLCULTURE™ PET membrane (5 µm pores)	**2000M12/510M503**	it4ip S.A., Louvain-la-Neuve, Belgium	-	-
Isopropanol ULSI	**Isopropanol**	MicroChemicals		

	**ULSI**	GmbH, Ulm,. Germany		
PDMS Silicone Elastomer Base and Curing Agent	**SYLGARD™ 184; 5498840000**	Biesterfeld Spezialchemie GmbH, Hamburg, Germany	-	-
TRAKETCH® PET 3.0 p S210x300 (PET membrane with 3 µm pores)	**030444**	SABEU GmbH & Co. KG, Northeim, Germany	-	-
**Cell culture media, supplements, on-chip media supply and stimulation agents**				
(−)-Isoproterenol hydrochloride	**I6504**	Sigma-Aldrich, St. Louis, MO, USA	1 mM – 100 mM in PBS^-	1 µM – 100 µM
21 GA stainless steel plastic hub dispensing needles	**400-3895 (Kahnetics - KDS2112P)**	RS Components GmbH, Frankfurt am Main, Germany	-	-
23 GA stainless steel plastic hub dispensing needles	**400-8272 (Kahnetics - KDS2312P)**	RS Components GmbH, Frankfurt am Main, Germany	-	-
Cytiva HyClone™ FetalClone™ II Serum (USA)	**10326762**	Thermo Fisher Scientific Inc., Waltham, MA, USA	-	-
Disposable syringe Injekt® With Luer-Lock fitting, 2 ml (from B. Braun)	**EP95.1**	Carl Roth GmbH + Co. KG, Karlsruhe, Germany		
DMEM, high glucose	**11965092**	Thermo Fisher Scientific Inc., Waltham, MA, USA	-	-
DMEM/F-12, no phenol red	**21041025**	Thermo Fisher Scientific Inc., Waltham, MA, USA	-	-
eBioscience™ Lipopolysaccharide (LPS) Solution (500X)	**00-4976-93**	Thermo Fisher Scientific Inc., Waltham, MA, USA	Further diluted to 100 µg/ml	100 ng/ml
Endothelial Cell Growth Media (ECGM)	**C-22010**	PromoCell GmbH, Heidelberg, Germany	-	-
Gentamicin	**15710064**	Thermo Fisher Scientific Inc., Waltham, MA, USA	10 mg/ml	0.1 mg/ml
HEPES (1M)	**15630056**	Thermo Fisher Scientific Inc., Waltham, MA,	1 M	10 mM

		USA		
Human IL-4	**130-093-920**	Miltenyi Biotec B.V. & Co. KG, Bergisch Gladbach, Germany	20 µg/ml in PBS^+	20 ng/ml
HyStem-C	**GS313**	CellSystems® GmbH, Troisdorf, Germany	-	-
Insulin solution, human	**I9278**	Sigma-Aldrich, St. Louis, MO, USA	10.8 mg/ml	65.1 nM
Lidocaine hydrochloride	**L5647**	Sigma-Aldrich, St. Louis, MO, USA	-	-
Penicillin-Streptomycin	**15140148**	Thermo Fisher Scientific Inc., Waltham, MA, USA	10,000 U/ml	100 U/ml
Recombinant Human GM-CSF	**300-03**	PeproTech Germany, Hamburg, Deutschland	10 µg/ml in autoclaved MilliQ	10 ng/ml
Rosiglitazone	**R2408**	Sigma-Aldrich, St. Louis, MO, USA	5 mM in DMSO	100 nM
TNF-α human	**SRP3177**	Sigma-Aldrich, St. Louis, MO, USA	100 µg/ml in PBS^+	20 ng/ml
TrypLE™ Select Enzyme (1X), no phenol red	**12563011**	Thermo Fisher Scientific Inc., Waltham, MA, USA	-	-
TYGON® tubing ND-100-80/	**5205508**	OMNILAB-LABORZENTRUM GmbH & Co. KG, Bremen, Germany	-	-
Versene Solution	**15040066**	Gibco	-	-
X-VIVO™ 15 Serum-free Hematopoietic Cell Medium	**BE02-060F**	Lonza Group AG, Basel, Switzerland	-	-
**Cell isolation reagents/MACS equipment**				
autoMACS Rinsing Solution	**130-091-222**	Miltenyi Biotec B.V. & Co. KG, Bergisch Gladbach, Germany	-	-
CD14 MicroBeads, human, 2 ml	**130-050-201**	Miltenyi Biotec B.V. & Co. KG, Bergisch Gladbach, Germany	-	-

Collagenase NB 4 Standard Grade	**S1745401**	Serva Electrophoresis GmbH, Heidelberg, Germany	-	0.13 U/ml
Dispase	**D4693**	Merck KGaA, Darmstadt, Germany	-	2 U/ml in PBS$^-$
Histopaque® 1077	**10771**	Merck KGaA, Darmstadt, Germany	-	-
LS+ MACS Column	**130-042-401**	Miltenyi Biotec B.V. & Co. KG, Bergisch Gladbach, Germany	-	-
MACS BSA Stock Solution	**130-091-376**	Miltenyi Biotec B.V. & Co. KG, Bergisch Gladbach, Germany	-	-
Red Blood Cell Lysis Solution (10x)	**130-094-183**	Miltenyi Biotec B.V. & Co. KG, Bergisch Gladbach, Germany	-	-
**Staining solution requirements**				
Antibody Diluent, Background Reducing	**S3022**	Agilent Technologies, Inc, Santa Clara, CA, USA	-	-
ROTI®Histofix 4 %	**P087.6**	Carl Roth GmbH + Co. KG, Karlsruhe, Germany	-	-
Saponin	**47036**	Sigma-Aldrich, St. Louis, MO, USA	-	0.2% (w/v)
Triton™ X-100	**X100**	Sigma-Aldrich, St. Louis, MO, USA	-	0.3% (v/v)
**Dyes**				
4′,6-Diamidino-2-phenyl-indol – dihydrochlorid (DAPI ready made solution)	**MBD0015**	Sigma-Aldrich, St. Louis, MO, USA	1 mg/ml	1-2 µg/ml
BODIPY™ 493/503 (4,4-Difluoro-1,3,5,7,8-Pentamethyl-4-Bora-3a,4a-Diaza-	**D3922**	Thermo Fisher Scientific Inc., Waltham, MA, USA	1 mg/ml (3.8 mM) in anhydrous DMSO	1-2 µg/ml (3.8 µM)

s-Indacene)				
**Conjugated antibodies**				
CD106 (VCAM-1) Antibody, anti-human, REAfinity™ (staining prior to fixation)	**130-104-164**	Miltenyi Biotec B.V. & Co. KG, Bergisch Gladbach, Germany	-	(1:10)
CD11c Antibody, anti-human, APC, REAfinity™ (staining after fixation)	**130-114-110**	Miltenyi Biotec B.V. & Co. KG, Bergisch Gladbach, Germany	-	(1:25)
CD309 (VEGFR-2) Antibody, anti-human, REAfinity™ (staining prior to fixation)	**130-117-984**	Miltenyi Biotec B.V. & Co. KG, Bergisch Gladbach, Germany	-	(1:25)
CD31 Antibody, anti-human, REAfinity™ (staining prior to fixation)	**130-110-807**	Miltenyi Biotec B.V. & Co. KG, Bergisch Gladbach, Germany	-	(1:25)
CD45 Antibody, anti-human, APC, REAfinity™ (staining prior to fixation)	**130-110-771**	Miltenyi Biotec B.V. & Co. KG, Bergisch Gladbach, Germany	-	(1:25 – 1:10)
eNOS Antibody, anti-human, APC, REAfinity™ (staining after fixation)	**130-106-840**	Miltenyi Biotec B.V. & Co. KG, Bergisch Gladbach, Germany	-	(1:10)
REA Control Antibody (S), human IgG1, REAfinity™	**130-113-434**	Miltenyi Biotec B.V. & Co. KG, Bergisch Gladbach, Germany	-	(1:25 – 1:10)
**Primary antibodies**				
Anti-Perilipin A antibody produced in rabbit	**P1998**	Sigma-Aldrich, St. Louis, MO, USA	1.2 mg/ml	12 µg/ml (1:100)
Monoclonal Mouse Anti-Human CD31, Endothelial Cell (Dako Omnis)	**GA61061-2**<br>**Clone JC70A**	Agilent Technologies, Inc, Santa Clara, CA, USA	-	1:50

Purified Mouse Anti-Human CD68	**556059 Clone Y1/82A (RUO)**	Becton Dickinson (BD), Franklin Lakes, NJ, USA	0.5 mg/ml	5 µg/ml (1:100)
Recombinant Anti-CD3 antibody [SP162]	**ab135372**	Abcam, Cambridge, UK	0.5 mg/ml	5 µg/ml (1:100)
Recombinant Anti-CD86 antibody [EPR21962]	**ab239075**	Abcam, Cambridge, UK	569 µg/ml	11.38 µg/ml (1:50)
Recombinant Anti-Mannose Receptor antibody [EPR6828(B)]	**ab125028**	Abcam, Cambridge, UK	180 µg/mL	3.6 µg/ml (1:50)
TREM2 Recombinant Rabbit Monoclonal Antibody (9H4L26)	**702886**	Thermo Fisher Scientific Inc., Waltham, MA, USA	0.5 mg/mL	5 µg/ml (1:100)
**Secondary antibodies**				
F(ab')2-Goat anti-Rabbit IgG (H+L) Cross-Adsorbed Secondary Antibody, Alexa Fluor 555	**A-21430**	Thermo Fisher Scientific Inc., Waltham, MA, USA	2 mg/ml	20 µg/ml (1:100)
Goat anti-Mouse IgG (H+L) Cross-Adsorbed Secondary Antibody, Alexa Fluor 546	**A-11003**	Thermo Fisher Scientific Inc., Waltham, MA, USA	2 mg/ml	20 µg/ml (1:100)
Goat anti-Mouse IgG (H+L) Cross-Adsorbed Secondary Antibody, Alexa Fluor 488	**A-11001**	Thermo Fisher Scientific Inc., Waltham, MA, USA	2 mg/mL	20 µg/ml (1:100)
**Isotype controls**				
Rabbit IgG Isotype Control	**31235**	Thermo Fisher Scientific Inc., Waltham, MA, USA	11.0 mg/mL	5.5 µg/ml (1:2000)
Recombinant Rabbit IgG, monoclonal [EPR25A]	**ab172730**	Abcam, Cambridge, UK	1.675 mg/ml	8.4 µl/ml (1:200)
**Tracers for localization and functionality readouts**				
BODIPY™ 500/510 C1, C12 (4,4-Difluoro-5-Methyl-4-Bora-3a,4a-Diaza-s-Indacene-3-Dodecanoic	**D3823**	Thermo Fisher Scientific Inc.,	4 mM in PBS^{+}	4 µM

Acid)		Waltham, MA, USA		
BODIPY™ FL C16 (4,4-Difluoro-5,7-Dimethyl-4-Bora-3a,4a-Diaza-s-Indacene-3-Hexadecanoic Acid)	**D3821**	Thermo Fisher Scientific Inc., Waltham, MA, USA	4 mM in PBS^+	4 µM
CellTracker™ Deep Red Dye	**C34565**	Thermo Fisher Scientific Inc., Waltham, MA, USA	1 mM in DMSO	2 µM in DMEM
Fluorescein diacetate	**F7378**	Merck KGaA, Darmstadt, Germany	1 mg/mL in acetone	0.135 mg/mL
Fluoresceinisothiocyanat-Dextran (3-5 kDa)	**FD4**	Sigma-Aldrich, St. Louis, MO, USA	10 mg/ml in PBS	100 µg/ml
Fluoresceinisothiocyanat-Dextran (40 kDa)	**FD40**	Sigma-Aldrich, St. Louis, MO, USA	10 mg/ml in PBS	100 µg/ml
Hoechst 33342 Solution	**62249**	Thermo Fisher Scientific Inc., Waltham, MA, USA	20 mM	20 µM
Low Density Lipoprotein from Human Plasma, Acetylated, DiI complex (DiI AcLDL)	**L3484**	Thermo Fisher Scientific Inc., Waltham, MA, USA	1 mg/ml	1 µg/ml
Propidium iodide	**P4170**	Merck KGaA, Darmstadt, Germany	1 mg/mL in PBS^-	0.027 mg/mL
**Assays for effluent readouts**				
CytoTox 96® Non-Radioactive Cytotoxicity Assay	**G1780**	Promega GmbH, Walldorf, Germany	-	-
Free Glycerol Reagent	**F6428**	Sigma-Aldrich, St. Louis, MO,	-	-

		USA		
Glycerol Standard Solution	**G7793**	Sigma-Aldrich, St. Louis, MO, USA	2.5 mg/ml	-
LEGENDplex™ Human Adipokine Panel (13-plex)	**740196**	BioLegend, Inc., San Diego, CA, USA	-	-
LEGENDplex™ Human Angiogenesis Panel 1 (10-plex)	**740697**	BioLegend, Inc., San Diego, CA, USA	-	-
LEGENDplex™ HU Th Cytokine Panel (12-plex) w/ VbP V02	**741028**	BioLegend, Inc., San Diego, CA, USA	-	-
**Devices used for chip fabrication and tissue characterization**				
Flow cytometer	**Guava easyCyte 8HT**	Merck KGaA, Darmstadt, Germany	-	-
Fluorescence microscope	**DMi8**	Leica Microsystems CMS GmbH, Wetzlar, Germany	-	-
Laser cutter with 10 W CO_2 laser	**VLS2.30**	Universal Laser Systems, Inc., Scottsdale, AZ, USA	-	-
Laser scanning microscope	**LSM 710**	Carl Zeiss Microscopy GmbH, Jena, Germany	-	-
Plasma unit	**Zepto One**	Diener electronic GmbH & Co KG, Ebhausen,	-	-
Plate reader	**Infinite® 200 PRO**	Tecan Trading AG, Männedorf, Switzerland	-	-
Syringe pump	**LA-190, 12-channel**	Landgraf Laborsysteme HLL	-	-

		GmbH, Langenhagen, Germany		
**Software**				
COMSOL Multiphysics®	**COMSOL Vers.5.5**	COMSOL AB, Stockholm, Sweden	-	-
CorelCAD	**Vers. 2020**	Corel Corporation, Ottawa, Ontario, Canada	-	-
Fiji	**Image J version 1.53c**		-	-
LEGENDplex Cloud-Based Data Analysis Software Suite (BioLegend)	**-**	BioLegend, Inc., San Diego, CA, USA	-	-
